# Development of the Swiss Database for dosing medicinal products in pediatrics

**DOI:** 10.1007/s00431-021-04304-8

**Published:** 2021-11-05

**Authors:** Romy Tilen, Dalibor Panis, Samuel Aeschbacher, Thomas Sabine, Henriette E. Meyer zu Schwabedissen, Christoph Berger

**Affiliations:** 1 SwissPedDose, Zurich, Switzerland; 2 Department of Infectious Diseases and Hospital Epidemiology, University Children’s Hospital Zurich, Zurich, Switzerland; 3 Infoserv, Evilard, Switzerland; 4 Federal Office of Public Health, Bern, Switzerland; 5 Biopharmacy, Department of Pharmaceutical Science, University Basel, Basel, Switzerland

**Keywords:** Children, Drug safety, Off-label use, Prescribing, Web-based software

## Abstract

In daily paediatrics, drugs are commonly used off-label, as they are not approved for children. Approval is lacking because the required clinical studies were limited to adults in the past. Without clinical studies, evidence-based recommendations for drug use in children are limited. Information on off-label drug dosing in children can be found in different handbooks, databases and scientific publications but the dosing recommendations can differ considerably. To improve safety and efficacy of drugs prescribed to children and to assist the prescribers, stakeholders in Swiss paediatrics started a pilot project, supported by the Federal Office of Public Health, with the aim to create a database, providing healthcare professionals with so called “harmonised” dosage recommendations based on the latest available scientific evidence and best clinical practice. A standardised process for dosage harmonisation between paediatric experts was defined, guided and documented in an electronic tool, developed for this purpose. As proof of principle, a total of 102 dosage recommendations for 30 different drugs have been nationally harmonised in the pilot phase considering the current scientific literature and the approval of the most experienced national experts in the field.

*Conclusion*: This approach paved the way for unified national dosage recommendations for children. Reaching the project’s milestones fulfilled the prerequisites for funding and starting regular operation of SwissPedDose in 2018. Since then, the database was extended with recommendations for 100 additional drugs.

**Table:** 

**What is Known:** *• Prescribing off-label is a common practice among paediatricians, as many drugs are still not authorised for use in children.* *• Some countries developed national drug formularies providing off-label dosage recommendations.*	
**What is New:** *• Comparison of published dosage recommendations in known drug handbooks and online databases show substantial differences and heterogeneity, revealing the need for harmonisation.* *• The design of a tool for standardised harmonisation of dosage recommendations, based on information collected on currently applied dosages, latest scientific evidence and the approval of experts.*

**What is Known:**

*• Prescribing off-label is a common practice among paediatricians, as many drugs are still not authorised for use in children.*

*• Some countries developed national drug formularies providing off-label dosage recommendations.*

**What is New:**

*• Comparison of published dosage recommendations in known drug handbooks and online databases show substantial differences and heterogeneity, revealing the need for harmonisation.*

*• The design of a tool for standardised harmonisation of dosage recommendations, based on information collected on currently applied dosages, latest scientific evidence and the approval of experts.*

## Introduction

Children have often been excluded from the dosage-finding process and from drug approval [1]. Accordingly, most of the drugs administered in paediatrics are not authorised for use in children and as a result, there is no dosage recommendation for children on the drug label. Legal efforts worldwide aim at facilitating the development, performance of studies and accessibility of medicines in paediatrics. They target to ensure that more child-friendly medicines are approved and made available on the market [2–5]. Even if these regulations have stimulated paediatric research and the number of new products with specific paediatric indications is encouraging, there is still an immense lack of high-quality information about medicines used for children. A recent survey on paediatric information in the summary of product characteristics (SmPC) of medicines currently available in Germany concluded that the new regulation did not significantly stimulate clinical studies with medicines of which the patent has already expired [6]. Importantly, prescribing a medication for a specific age group or for a specific disorder not covered by the terms of its marketing authorisation and, therefore, not in accordance with the SmPC is called off-label use [7]. The proportion of off-label use in children varies, depending on factors such as age, health care setting and quantity of prescribed drugs. A survey in a Swiss university hospital revealed a proportion of off-label and unlicensed use in the inpatient sector of about 50% [8]. The reported numbers are referring to the total number of prescriptions in the investigated sector which is comparable with reports on off-label/unlicensed use for hospitalised children in other countries [9–11] and has not improved since the paediatric regulation [12, 13]. Without another choice, off-label use is a widespread and common practice for paediatrician [14, 15], without implying improper or illegal drug use [16]. Importantly, off-label use does not exclude that there is extensive clinical experience and also data for the intended use [17]. For some drugs, there is recent scientific data of high quality and ample experience for the dosage used in practice which may deviate from the information in the SmPC [18]. In addition, information in drug labels, such as warnings or contraindications for use in children and adolescents has in most cases only legal, but no medical meaning [6, 19]. On-label does not necessarily mean that it is based on current scientific evidence, as such a change in the SmPC has to be submitted by the marketing-authorisation holder [20]. Thus, off-label prescription is the paediatricians’ everyday practice, while following the label may not always be appropriate in children.

In 2008, 1 year after the introduction of the paediatric regulation in Europe, the Swiss Confederation recognised the unsatisfactory situation within paediatric drug therapy in Switzerland [21]. In particular, serious adverse drug reactions (ADRs) occurred three times more often in children than in adults [22–24]. A considerable proportion of these ADRs were due to mistakes in prescription, including incorrect dosages. The prescription of drugs should be based on the best available evidence, including information gathered in peer-reviewed scientific studies. In paediatrics, this information is commonly gathered by experts in academia (university hospitals), but it often does not find its way into the SmPCs’ marketing authorisation [25, 26].

The recognition of the Swiss federal government was in accordance with the call of Swiss paediatricians that the systematic collection and transparent provision of scientific data and clinical experience in paediatrics is of central importance [21]. This led to the decision of the federal government to extend the Therapeutic Products Act (TPA). This extension enabled the introduction of a national database for the collection, evaluation, harmonisation and publication of data relating to the prescription, supply and use of medicinal products in paediatrics [27].

Stakeholders in Swiss paediatrics launched the pilot project that we present here. Its overall aim was the development of this database to provide healthcare professionals with national harmonised dosage recommendations. Accordingly, the milestones of the project were the design of a web-based tool for the harmonisation of dosage recommendations for children in Switzerland, based on the information collected on currently applied dosages, latest scientific evidence from the literature, and the clinical experience and approval of experts and the publication of the results in a web application accessible to healthcare professionals free of costs in Switzerland and worldwide.

## Methods

### Collection of drug consumption data

The project started with a collection of drugs used in 8 contributing children’s hospitals in Switzerland, in order to decide which drugs are the most commonly used and should be harmonised first.

Data on drug consumption were collected from the hospital pharmacies including the regular consumption of all patients and all departments within the hospitals over a year. The lists were summarised and limited to 40 drugs. This “Top 40 list” was the basis for the collection of in-house dosage recommendations for these drugs from the participating hospitals.

### Online platform to track the harmonisation process

In order to define harmonised dosage recommendations for off-label drug use in Switzerland, a standardised harmonisation process was developed in which the dosage recommendations currently used for prescriptions in the hospitals were compiled, compared with the current scientific literature and the results assessed and approved among experts. In cooperation with the IT service provider Infoserv (Evilard, Switzerland), an online platform — The Harmonisation Tool — was designed to guide the process and to document discussions and decisions, so that all steps are traceable at any time.

**Fig. 1:**
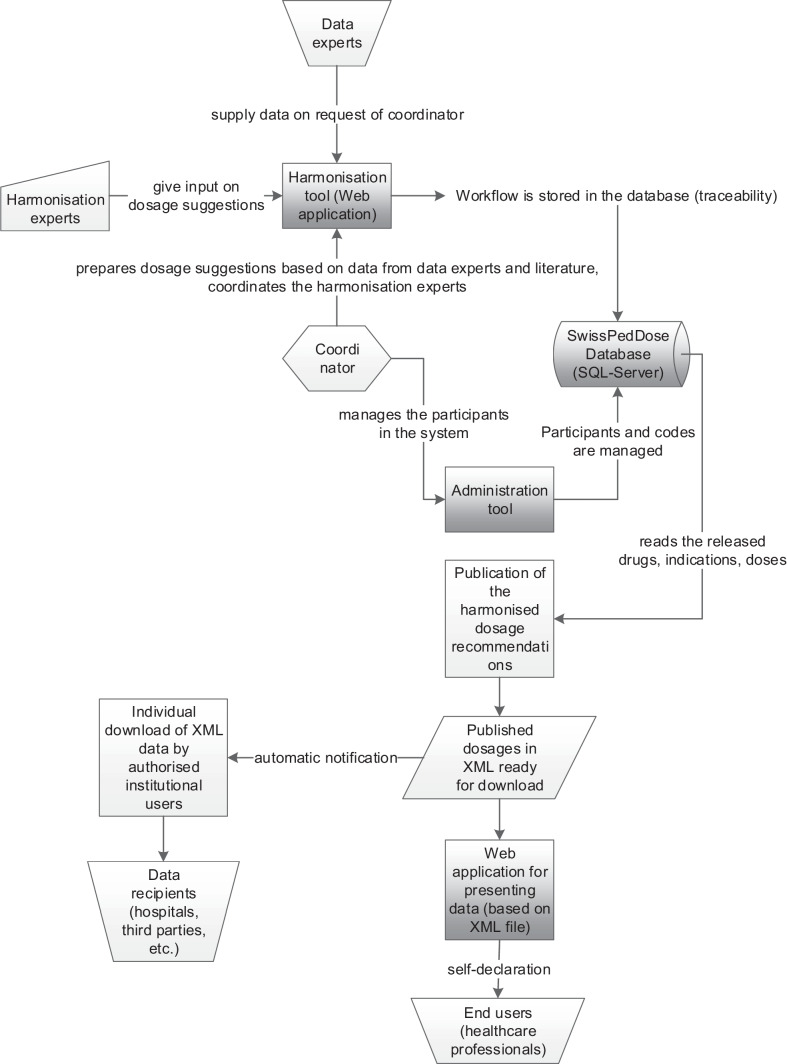
Developed tools. The harmonisation process is performed in the web-based harmonisation tool. The complete workflow with every access to and entry of data is stored in an SQL database. Role and personnel information of participants (data and harmonisation experts, coordinators) and used codes (e.g. indications, substances, routes of administration) can be managed in the separated administration tool. The dosage recommendations authorised for publication are exported as XML file. The XML data can be imported in the SwissPedDose web application or downloaded and used from registered data recipients

The harmonisation tool as a test and productive version can be accessed online by the project participants. The data entered into the harmonisation tool are stored within a SQL database. The eventually harmonised data is automatically published in XML format and imported in a web application that can be accessed by health care professionals (Fig. 1). In a separate administration tool, the roles of the harmonisation participants and all codes (indications, routes of administration, units, ATC codes, remarks etc.) can be managed by the coordinating pharmacists (coordinator).

### Operational project team

The team consists of data experts, harmonisation experts and a coordinator. The requirements for the experts were defined before the recruitment. They were delegated by their clinic directors, approved by the project manager and finally had to undergo training before they were able to participate in the harmonisation process. From each of the 8 hospitals, 1 hospital pharmacist (data expert) with access to the internal dosing guidelines joined the project team, as well as 3 senior paediatricians (harmonisation experts) with the most comprehensive professional experience in one of the following specialties: infectious diseases, neonatology and general paediatrics. Three expert groups of 8 harmonisation experts were formed and each group was led by a designated coordinator.

### Process of data collection

**Fig. 2:**
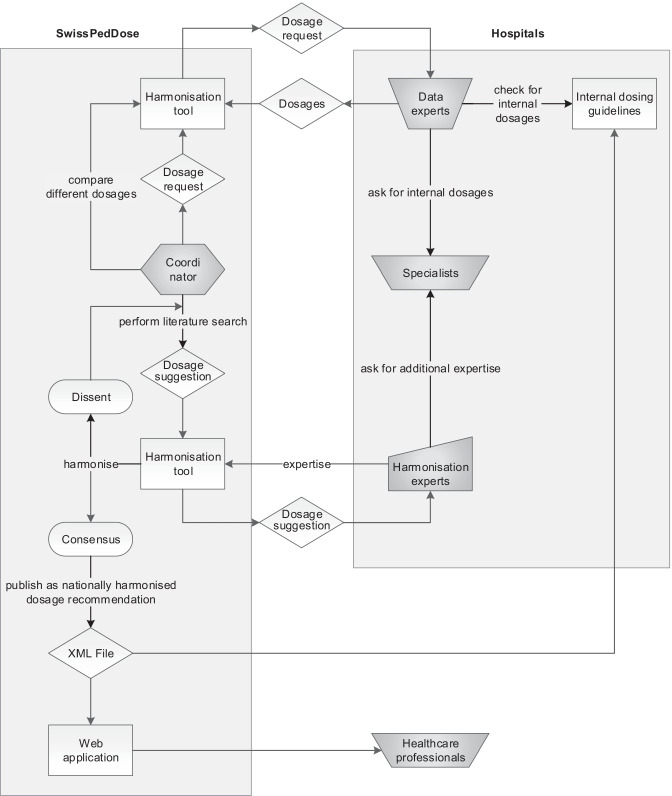
Schematic depicting the standardised harmonisation process. This process includes the gathering of data, their evaluation and consensus finding. Each step is tracked and digitalised in the harmonisation tool

In order to optimise the data collection, evaluation and consensus finding, a standardised process was developed for the data experts, the 3 harmonisation expert groups and the coordinators. The process for dosage harmonisation (Fig. 2) foresees that the coordinators request electronically the internal dosage recommendations and its underlying references from the 8 data experts. The request is limited to one drug-indication pair supplemented with the route of administration (e.g. paracetamol-fever-intravenous route) and the age and weight categories, if applicable. The data experts analyse the internal dosing guidelines and check with in-house specialists where necessary. The data experts send the in-house dosage recommendation via the harmonisation tool to the coordinator. If at least 4 of 8 hospitals are using the drug for this indication and route of administration, the coordinator starts the harmonisation round and performs a literature review.

### Literature review

A semi-structured literature review was conducted to find recent dosage recommendations in the literature and to compare the findings with the collected dosing guidelines from the participating hospitals.

The literature review started by analysing the “Therapeutic indications” and “Posology” sections of the drugs’ newest SmPCs approved and published by the Swiss Agency for Therapeutic Products, the European Medicine Agency or the Food and Drug Administration, respectively [28–30]. For that the drug formulation matching the route of administration was selected. It was checked whether the drug is licensed for any age group within the paediatric population, and if so, the respective dosage recommendation was included. Then peer-reviewed literature sources were consulted. At first, well-known international and national referenced drug handbooks and online databases were searched for the drug and its dosages for each indication [31–44]. Additionally, we searched for the selected drug filtered by age (newborn, child), and if possible, the indication/route of administration of interest on websites of medical societies, in the PubMed database, and in the Cochrane Library for national and international consensus guidelines and systematic reviews. Original literature referenced in these literature sources was analysed, and paediatric drug dosage information contained therein was taken into account.

The information found is gathered in a PDF summary containing all published dosage recommendations, the related references, and if applicable, the corresponding age and weight categories for which the recommendations are made.

### Process of harmonisation

Subsequently, the coordinator made a dosage suggestion to be discussed within the respective expert group (Fig. 2). This dosage suggestion results from the highest evidence (e.g. randomised controlled trial or guideline of a medical society) and comparison of practical aspects (e.g. administration modalities and dosage frequencies in order to achieve the best possible compliance) of all dosage recommendations gathered.

The dosage suggestion is entered into the harmonisation tool, the literature summary is attached, validated by a second coordinator and sent to the 8 harmonisation experts. If they agree, they accept it. If they disagree, they can directly mutate the suggestion and have to justify their changes referencing the related evidence and/or scientific literature. All experts are able to view the changes requested along with the respective comments of their colleagues, like in an interactive chatroom. If necessary, the coordinator adapts the dosage suggestion based on the feedback and its scientific evidence. The process is closed when all 8 harmonisation experts in the group agree with the suggestion and a consensus is reached. The consensus and its entry in the harmonisation tool is double checked by a second coordinator and then published as the national harmonised dosage recommendation on the SwissPedDose web application.

## Results

### Drug consumption data and collection of in-house dosage recommendations

The collected drug consumption data, resulted in a “Top 40 list” of the most frequently used drugs in Swiss children’s hospitals (Table 1).

**Table 1:** Summarised top 40 drug list, alphabetically sorted. Shown is the summary of drug consumption data gathered and submitted by the pharmacies of 8 children’s hospitals in Switzerland, limited to 40 drugs after processing. For each listed substance, the number of reporting hospitals is stated. ^†^harmonised during pilot project; ^‡^not licensed for children in Switzerland

**Substance**	**ATC code**	**Drug groups **[55]	**Number of reporting hospitals**
Acyclovir^†^	J05AB01	ANTIVIRALS	4/8
Amoxicllin^†^	J01CA04	ANTIBACTERIALS	8/8
Amoxicillin-clavulanate^†^	J01CR02	ANTIBACTERIALS	7/8
Betamethasone^†^	H02AB01	CORTICOSTEROIDS	4/8
Ceftazidime^†^	J01DD02	ANTIBACTERIALS	5/8
Ceftriaxone^†^	J01DD04	ANTIBACTERIALS	5/8
Cefuroxime^†^	J01DC02	ANTIBACTERIALS	4/8
Clonidine^‡^	C02AC01	ANTIHYPERTENSIVES	3/8
Caffeine citrate^‡^	N/A	N/A	5/8
Dexamethasone^†^	H02AB02	CORTICOSTEROIDS	6/8
Diclofenac^†^	M01AB05	ANTI-INFLAMMATORY AND ANTI-RHEUMATIC PRODUCTS	4/8
Epinephrine^†^	C01CA24	CARDIAC THERAPY	5/8
Esomeprazole	A02BC05	DRUGS FOR ACID-RELATED DISORDERS	3/8
Fentanyl^†^	N01AH01	ANESTHETICS	6/8
Fluconazole^†^	J02AC01	ANTIMYCOTICS	3/8
Furosemide^†^	C03CA01	DIURETICS	6/8
Gentamicin^†‡^	J01GB03	ANTIBACTERIALS	3/8
Heparin^†^	B01AB01	ANTITHROMBOTIC AGENTS	7/8
Hydrochlorothiazide	C03AA03	DIURETICS	4/8
Hydrocortisone	H02AB09	CORTICOSTEROIDS	3/8
Ibuprofen^†^	M01AE01	ANTI-INFLAMMATORY AND ANTIRHEUMATIC PRODUCTS	8/8
Infliximab^†^	L04AB02	IMMUNOSUPPRESSANTS	5/8
Macrogol	A06AD15	DRUGS FOR CONSTIPATION	4/8
Meclozine	R06AE05	ANTIHISTAMINES	3/8
Mefenamic acid	M01AG01	ANTI-INFLAMMATORY AND ANTIRHEUMATIC PRODUCTS	4/8
Meropenem^†^	J01DH02	ANTIBACTERIALS	3/8
Metamizole	N02BB02	ANALGESICS	4/8
Methylprednisolone	H02AB04	CORTICOSTEROIDS	3/8
Metronidazole^†^	P01AB01 and J01XD01	ANTIPROTOZOALS and ANTIBACTERIALS	5/8
Midazolam^†^	N05CD08	PSYCHOLEPTICS	7/8
Morphine^†^	N02AA01	ANALGESICS	7/8
Omeprazole	A02BC01	DRUGS FOR ACID-RELATED DISORDERS	4/8
Ondansetron^†^	A04AA01	ANTI-EMETICS and ANTINAUSEANTS	7/8
Paracetamol^†^	N02BE01	ANALGESICS	8/8
Prednisolone	H02AB06	CORTICOSTEROIDS	4/8
Propofol	N01AX10	ANESTHETICS	3/8
Propranolol	C07AA05	BETA BLOCKING AGENTS	3/8
Spironolactone^†^	C03DA01	DIURETICS	6/8
Trimethoprim-sulfamethoxazole^†^	J01EE01	ANTIBACTERIALS	3/8
Vancomycin^†^	A07AA09 and J01XA01	ANTIDIARRHEALS, INTESTINAL ANTI-INFLAMMATORY/ANTI-INFECTIVE AGENTS and ANTIBACTERIALS	3/8

The collection of in-house dosage recommendations for these drugs from the participating hospitals revealed that the majority of the children’s hospitals in Switzerland have their own dosage databases or booklets, which are historically grown and are often lacking information on their origin. Accordingly, there is heterogeneity with sometimes considerable differences in dosage recommendations. In order to exemplify this fact, Table 2 is providing a summary of in-house data gathered for the antibiotic gentamicin. Information provided was limited to general dosing what usually relates to severe infections and to the age group from ≥ 1 month to < 12 years of age. Importantly, gentamicin is an antibiotic known for its potential side effects where therapeutic drug monitoring is required due to the clear link between drug concentrations in the blood and occurrence of nephro- or ototoxicity [45]. The list reveals that there is the need for a harmonisation.

**Table 2:** Summary of the in-house dosage recommendations of intravenous gentamicin, extracted from the national database SwissPedDose; ^†^indicates where a 24-h dosing interval applies; ^‡^indicates where an 8-h dosing interval applies. *n.r.* no in-house recommendation reported

**Hospital**	**Dosage** (mg/kg/dose)	**Trough level** (mg/L)	**Peak level** (mg/L)
1	3.0–7.5^†^	0.5–2.0	n.r
2	No gentamicin use	n.r	n.r
3	7.5^†^	0–2.0	5–10
4	5.0–7.0^†^	≤ 1.0	n.r
5	5.0–7.5^†^ (max. daily dose 500 mg/dose^†^)	< 1.0	6–8
6	Moderate infection: 1.0^‡^ Severe infection: 2.5^‡^	< 0.6–2.0	n.r
7	7.5^†^	< 1.0	n.r
8	< 33 kg body weight: 7.5^†^ ≥ 33 kg body weight: 250 mg/dose^†^	< 2.0	n.r

### Literature review

The search for paediatric dosage information in internationally known and well-established drug handbooks and online databases also reveals that these evidence-based/referenced sources contain differing dosage recommendations for the same indication. One illustrative example is shown in Table 3, where we again summarise the dosage recommendations for gentamicin but this time published in the literature references listed.

**Table 3:** Summary of dosage recommendations extracted from different literature sources for intravenous gentamicin; *n.r.* no trough level reported

**Literature**	**Dosage** (mg/kg/dose)	**Dosing interval**	**Trough level** (mg/L)
BNF for Children 2016–17	7.0	q24h	n.r
Kinderformularium, accessed 24.01.2017	7.0	q24h	n.r
Nelson’s Pediatric Antimicrobial Therapy, 22nd Edition 2016	3.0–7.5	q8–24h	n.r
RedBook®, 30th Edition 2015	2.0–2.5 5.0–7.5	q8h q24h	n.r
Shann, F., Drug Doses, 16th Edition 2014	Initial 8.0, then 6.0 (1 w–10 y) Initial 7.0, then 5.0 (> 10 y) Max. daily dose 240–360 mg/dose	q24h	< 1.0
SmPC Gentamicin-ratiopharm®, accessed 24.01.2017	4.5–7.5 (1 m–3 y) 3.0–6.0 (3–17 y)	q12–24h	< 1.0 (q24h) < 2.0 (q12h)
UpToDate®, accessed 24.01.2017	2.0–2.5 4.5–7.5	q8h q24h	n.r

In this example of intravenous gentamicin, the dosage suggestion made by the coordinator after compiling the internal dosage recommendations of the 8 children’s hospitals, performing the literature review and comparing the results was 7.5 mg/kg/dose q24h with a maximum daily dose of 500 mg/dose and a trough level of < 2 mg/L. After sending the dosage suggestions to the 8 harmonisation experts specialised in the field of paediatric infectious diseases, they disagreed on the indication of maximum daily dose because evidence was scarce and the availability of dosage adjustment by therapeutic drug monitoring. With agreement of all experts after the second round of harmonisation, the coordinator published the dosage recommendation without a maximum daily dose.

### Dosage recommendations

During the pilot project, a total of 177 data requests for 36 different drugs were sent to the data experts. One hundred forty-eight (83.6%) of the requests were answered by all 8 data experts. For 105 of the answered tasks, where at least 4 of 8 hospitals provided an internal dosage recommendation, a literature review was performed by the coordinator, and a dosage suggestion was prepared for the harmonisation experts. Of these, 27 (26%) cases were harmonised in the first round. For 71 (68%) cases, 2–3 harmonisation rounds were required to reach consensus. In 4 (4%) cases, more harmonisation rounds were needed. Only in 3 (3%) cases consensus was not reached. These 3 cases involved the following drugs: ceftazidime (cystic fibrosis, intravenous continuous infusion), ceftriaxone (Lyme disease, intravenous/intramuscular injection), and amoxicillin and beta-lactamase inhibitor (urinary tract infection, oral application).

By the end of the pilot project, a total of 102 national harmonised dosage recommendations were published. These are concerning 30 different drugs in the fields of paediatric infectious diseases, neonatology and general paediatrics. Twenty-six of these drugs were from the top 40 list and 4 additional drugs (amikacin, potassium canrenoate, nalbuphine and teicoplanin) have been harmonised upon request by the stakeholders.

### Publishing process and quality management

The harmonised dosage recommendations can be exported and downloaded as complete XML dataset by institutional users (e.g. hospitals or other authorised third parties) free of charge. If a new XML file is published, the registered users automatically receive a notification. The access to the web application is limited to healthcare professionals upon a mandatory self-declaration.

The harmonisation work was standardised and described in a quality management manual. The manual defines and explains the harmonisation process as well as the technical process of harmonisation in the harmonisation tool. Standard operating procedures (SOPs) for each of the process steps (e.g. literature review) were issued and applied. In addition to the SOPs, the manual contains personnel documents, which define the tasks, profiles and background of each participating person and describes the cooperation of the individual actors in the harmonisation process.

The dosage recommendations are re-evaluated regularly (i.e. when a request is received or at least every 4 years) to ensure that they remain up to date. For example a review of dosage recommendations for intravenous gentamicin dosing has recently resulted in an adjustment of trough level to < 1 mg/L [46].

## Discussion

Here, we describe the pilot project of the SwissPedDose database, in which we applied a standardised process for the national harmonisation of dosage recommendations for paediatric patients. The overarching goal behind this initiative is to improve quality of dosage recommendations and pharmacotherapy of children by making recommendations widely available that are based on the latest scientific evidence and approved by respective clinical experts. This we see as most relevant as dosages by drug label in practice are not yet available or frequently enough not optimally adapted to children [47].

The development of an IT tool to guide and track all process steps and a web application to provide the recommendations to all healthcare professionals are the good result and substantial merit of this project. The tools allow traceability in line with quality assessment needs. Importantly, the interprofessional approach involved both hospital pharmacists and senior paediatricians of the largest children’s hospitals in Switzerland, thereby strengthening acceptance and support of the project objectives which is of utmost relevance for implementation. In all of the participating hospitals, the harmonisation process led to an update of previously used in-house dosage recommendation by implementation, indeed illustrating a quality improvement step. However, the involvement of 8 hospitals and the very high degree of traceability inevitably led to a slow and complex process that has resulted in a comparatively small number of harmonised dosage recommendations at this first stage.

We expect that implementation and extension of the harmonised and regularly re-evaluated dosage recommendations simplify the dosage-finding and prescription of medicinal products in children and will contribute to an increased efficacy and safety in paediatric drug therapy. This will have to be tested in future studies. To ensure implementation after the pilot phase, all participating children’s hospitals have signed a letter of intent to integrate the SwissPedDose dosage recommendations into their clinical information systems. In the meantime, all these children’s hospitals have implemented this goal.

A limitation of SwissPedDose is that for practical reasons the published dosage recommendations are in mg/kg body weight or mg/m^2^ body surface what partly leaves age-dependant pharmacokinetic variability unconsidered [48]. The individual dosage must be calculated what bears the risks of dosing errors [23]. In some cases, where appropriate dosing information for special drugs, indications and age categories is lacking, further data is needed to optimise dosage recommendations.

It is important to identify and communicate these needs as research questions to the research community, to eventually induce appropriate clinical trials or physiologically based pharmacokinetic modelling. We were following this approach for the antibiotic gentamicin [49, 50].

With the established harmonisation process, it was possible to achieve national standardisation for 102 off-label dosage recommendations of 30 various drugs for children. Paediatric drug dose information sources from other countries such as Kinderformularium (NL), Lexicomp® (USA) and BNF for Children (UK) are comparable and follow a similar approach [35, 51, 52]. The Dutch group managing the Kinderformularium recently established the BRAvO framework, which offers a structured assessment of benefits and risks of off-label drug use in children [53]. We expect that the proposed benefit/risk assessment will further promote safe pharmacotherapy, when applied to the SwissPedDose dosage recommendations.

The development of a dedicated electronic tool to guide and trace the harmonisation process is unique for Switzerland. The tool could be useful for international harmonisation and the establishment of a European formulary. This is worth consideration, because there is no international consensus on which dose to adopt. Consensus may be difficult to achieve, as compilation varies by the review of available scientific evidence, and drug use is based on national clinical experience, when studies are lacking [54].

The project described herein was successful, and the milestones set at the beginning of the project were reached, so that it was possible to start a regular operation of the database from April 2018. At the time of writing, 448 dosage recommendations on 134 drugs are accessible at https://swisspeddose.ch/database.

## Data Availability

The data that support the findings of this project are available from the corresponding author upon request.

## Abbreviations

ADR: Adverse drug reaction
FOPH: Federal Office of Public Health
SmPC: Summary of Product Characteristics
SOP: Standard operating procedures
TPA: Therapeutic Products Act

## Declarations

### Disclaimer

The revised Therapeutic Products Act and the implementing provisions define the responsibilities of the SwissPedDose association and the FOPH respectively in the implementation of the national database.
